# Concepts for Developing Physical Gels of Chitosan and of Chitosan Derivatives

**DOI:** 10.3390/gels4030067

**Published:** 2018-08-09

**Authors:** Pasquale Sacco, Franco Furlani, Gaia de Marzo, Eleonora Marsich, Sergio Paoletti, Ivan Donati

**Affiliations:** 1 Department of Life Sciences, University of Trieste, Via Licio Giorgieri 5, I-34127 Trieste, Italy; franco.furlani@phd.units.it (F.F.); gaia.dema92@libero.it (G.d.M.); paolese@units.it (S.P.); 2 Department of Medicine, Surgery and Health Sciences, University of Trieste, Piazza dell’Ospitale 1, I-34125 Trieste, Italy; emarsich@units.it

**Keywords:** chitosan, gel, physical interactions, gelling mechanisms, tissue engineering, drug delivery, chitosan-derivatives, Chitlac

## Abstract

Chitosan macro- and micro/nano-gels have gained increasing attention in recent years, especially in the biomedical field, given the well-documented low toxicity, degradability, and non-immunogenicity of this unique biopolymer. In this review we aim at recapitulating the recent gelling concepts for developing chitosan-based physical gels. Specifically, we describe how nowadays it is relatively simple to prepare networks endowed with different sizes and shapes simply by exploiting physical interactions, namely (i) hydrophobic effects and hydrogen bonds—mostly governed by chitosan chemical composition—and (ii) electrostatic interactions, mainly ensured by physical/chemical chitosan features, such as the degree of acetylation and molecular weight, and external parameters, such as pH and ionic strength. Particular emphasis is dedicated to potential applications of this set of materials, especially in tissue engineering and drug delivery sectors. Lastly, we report on chitosan derivatives and their ability to form gels. Additionally, we discuss the recent findings on a lactose-modified chitosan named Chitlac, which has proved to form attractive gels both at the macro- and at the nano-scale.

## 1. Introduction

The term “gel” is usually associated with highly hydrated networks in which two components are present in different proportion, i.e., the solvent—the one prevailing in mass—and the polymeric solute; typically, the latter ones are either natural or synthetic macromolecules able to properly retain a large amount of the former component. Although this simple description is reasonably valid, it is complicated to define gels in detail. One of the safest ways to categorize pure viscous liquids from strong elastic gels is to assess their mechanical behavior, specifically undergoing the system to strain under oscillatory conditions for a large range of frequencies using rheometry as the analytical tool [1]. For limiting angular frequency, ω, namely ω→0, viscous liquids will display the storage modulus, G’, lower than the loss modulus, G’’; they typically scale with the following laws: $G\prime \approx \omega^{2}$ and G″≈ω. Conversely, strong gels will show G′ >> G″ with almost constant values over a large interval of frequencies, thus behaving as elastic solids.

The use of gels is widespread and growing, especially in the biomedical sector [2,3,4]. For instance, tissue engineering and regenerative medicine approaches require suitable matrices, described as “scaffolds”, which should recapitulate the physical milieu of the extracellular microenvironment. Hence, the fabrication of macro-gel networks endowed with peculiar composition and mechanical behavior would be beneficial for cellular anchoring, colonization and metabolic activity. Moreover, the application of micro- and nano-gels is attractive especially for drug delivery purposes. Obviously, this type of material should be biocompatible and, in general, biodegradable in due time, without eliciting any immune response if implanted or administered. Naturally-derived polymers, such as polysaccharides, are nowadays exploited for the fabrication of artificial extracellular matrix (ECM)-like macro-networks and micro- and nano-systems, since they possess most of the above-mentioned properties. In this respect, chitosan represents a very attractive biopolymer both for tissue engineering and drug delivery purposes [5,6].

The present review article summarizes the basilar gelling concepts for developing chitosan and chitosan-derivatives gels. Although the fabrication of chitosan-based gels may be achieved through different strategies, herein we focus the attention exclusively on those materials synthesized by exploiting physical interactions. The potential applications of such chitosan gels in the biomedical field are discussed as well. In the last section, we report on a lactose-modified chitosan produced by our research group, named Chitlac, or CTL, which recently proved to form both macro- and nano-gels, with very interesting physical and biological features.

## 2. An Overview of Chitosan Physical/Chemical Properties and Biological Activities

**Figure 1:**
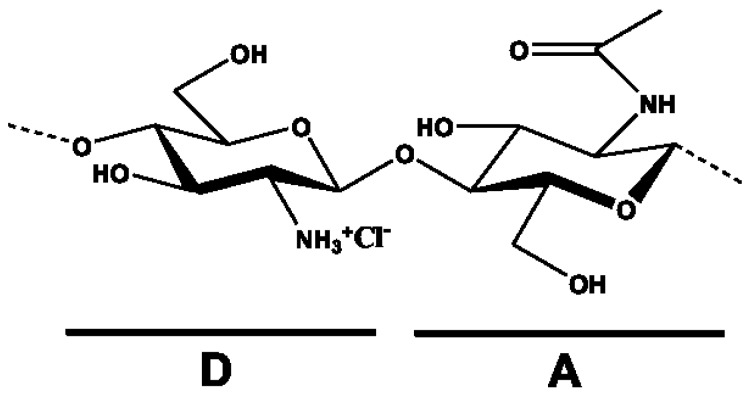
Schematic structure of chitosan: glucosamine (D) and *N*-acetyl-glucosamine (A) units.

Chitosans (CH) are considered as a family of binary heteropolysaccharides composed of β-1 → 4 linked 2-acetamido-2-deoxy-β-d-glucopyranose (GlcNAc, the “acetylated”, i.e., the A unit) and 2-amino-2-deoxy-β-d-glucopyranose (GlcNH_2_, the “deacetylated”, i.e., the D unit) residues, present in different relative proportion and sequence along the chain (Figure 1). Generally, D sugars are higher in number with respect to the A ones. Chitosans typically show an acetylation degree in the range 0–70%. As a general consideration, both building units are randomly distributed along the polymer chain, with a quasi-ideal Bernoullian arrangement of A and D sugars.

Chitosan derives mainly from chitin—the second most abundant polysaccharide on Earth—present in the outer skeleton of Arthropoda, or from the cell walls of certain fungi [7]. When chitin is used as the raw material, both homogeneous and heterogeneous deacetylation processes using alkaline conditions allow forming chitosans with a different fraction of A units (F_A_), [8] at the same time limiting chain depolymerization. Deacetylation processes under alkaline conditions are, in fact, well tolerated with respect to acidic ones, the latter leading to a severe cleavage of glycosidic linkages. Oppositely, homogeneous re-*N*-acetylation processes of highly deacetylated chitosans using hydroalcoholic media allow modulating the polymer chemical composition, producing chitosans with different F_A_ [9,10,11].

Concerning solution properties, chitosans behave as amphiphilic polymers because of the more hydrophobic nature of A sugars and the more hydrophilic character of D units. As a general consideration, all chitosans are water-soluble in acidic conditions due to the protonation of amino groups at the C2 position: in these conditions, chitosans behave as polyelectrolytes with a linear charge density which depends mainly on both F_A_ and pH. The pK_a_ was found to span from around 6.3 to 7.2 (α=0.5), depending on the F_A_ of chitosan [9], albeit other works reported no significant dependence of pK_a_ on F_A_ [12,13].

The solubility of chitosans at neutral pH and above is somewhat limited when typical commercial chitosans are considered. As an example, a medium molecular weight chitosan with F_A_ < 0.3 would precipitate when pH is increased toward neutrality. Two characteristics mainly contribute to chitosan solubility at neutral and slight alkaline pH: the molecular weight and the value of F_A_. A highly deacetylated chitosan with F_A_ ~ 0 begins to be soluble at neutral pH if the molecular weight is reduced to the point of reaching a degree of polymerization, DP < 10 [14]. On the other hand, medium/high molecular weight chitosans are soluble at neutral pH only if the acetylation degree is approximately 40% and above, typically in the range 0.4 < F_A_ < 0.6 [15,16].

From the biological point of view, chitosans are considered as low-toxic, non-immunogenic, and biodegradable polysaccharides. In the latest years, chitosans were extensively studied in terms of antibacterial properties and bioactivity. Different papers report on antibacterial properties. Zheng et al. demonstrated that the antimicrobial activity of chitosans can be modulated by varying polymer molecular weight and concentration [17]. A recent study by Younes et al. reported that the antibacterial activity of chitosan against Gram-negative bacteria increases with decreasing F_A_, molecular weight and pH [18]. When Gram-positive bacteria are considered, F_A_ and pH seemed to have a minor influence. The overall results seem to suggest that electrostatic interactions between the negative bacterial cell wall and positively-charged -NH_3_^+^ groups are involved in its antibacterial behavior, albeit hydrophobic and chelating effects cannot be excluded [19]. With respect to its antifungal activity, the influence of molecular weight and F_A_ of chitosan was correlated to the particular type of fungus considered [18]. Chitosans were also studied in terms of anti-HIV-1, antitumor and antioxidant activities, as documented in a recent review article [20]. Given all aforementioned properties, chitosans find application in very different sectors, spanning from biomedical—e.g., for the production of biomaterials for tissue engineering and drug delivery purposes—to water treatment and the food industry.

## 3. Physical Gelation of Chitosan

Unlike other marine polysaccharides such as alginates and carrageenans, chitin—and its *N*-deacetylated derivative, chitosan—are not present in the gel form in nature, where it plays mostly a structural role forming very strong fibrillar structures. Different research groups have proposed in recent years challenging strategies to promote the sol-gel transition of *N*-deacetylated chitins (chitosans). It is widely accepted that two ways can be essentially undertaken to form chitosan gels, i.e., by means of “physical” or “chemical” reticulation processes. In the latter case, covalent bonds among chains are exploited to form permanent networks. Small molecules, as well as glutaraldehyde, diglycidyl ether, diisocyanate or diacrylate are typically used to prepare chemical gels of chitosan. However, the well-known toxicity of most of such molecules represents a serious concern. Alternative strategies were proposed to limit this flaw, while promoting polymer-polymer reticulation. The authors of the present review refer elsewhere for a thorough description of the common concepts for synthesizing chitosan gels through chemical processes [6,21,22].

Physical chitosan gels are perhaps the most appealing systems, due to, in general, lower toxicity, the possibility of tuning both extent and rate of gel swelling, degradation and mechanical behavior. As a general consideration, however, physical chitosan gels are characterized by lower mechanical properties with respect to chemical gels. The sol/gel transition in physical gels is potentially achievable by varying physical/chemical parameters, such as temperature, pH, and ionic strength, or by the addition of suitable counterions. Electrostatic and hydrophobic interactions together with hydrogen bonds cooperate for the formation of entangled networks. As to ionic interactions, pH and ionic strength play a key role in gel formation. Chitosans behave as polyelectrolytes depending on the pH value of the solution. It means that chitosans can interact with ions, small molecules or macromolecules bearing opposite charges in a typical pH range of 4–6. The kinetics of self-assembling is generally very fast, albeit it can be easily tuned by increasing the ionic strength or crosslinker concentration [23]. Although the formation of chitosan gels in the micro/nano-size range is now relative easy to achieve via coacervation methodologies, more demanding processes are required for fostering the controlled gelation of chitosan at the macro-scale. As to the role of hydrophobic interactions and hydrogen bonding, additional variables affect the gelation of chitosan, such as polymer chemical composition (namely, the degree of acetylation, as a parameter tuning both types of interaction) and the use of co-solvents.

## 4. Chitosan Gels without External Crosslinkers

One of the simplest ways to produce physical gels of chitosan—without the use of any external gelling agent—is to precisely modulate the polymer chemical composition. Hirano et al. reported the formation of a true physical chitosan gel via controlled re-*N*-acylation processes of an almost completely deacetylated chitosan [24]. Specifically, chitosan solutions were treated with large amounts of acetic anhydride as acetyl group donor. Authors claimed that the excess of acetic anhydride was pivotal for the gel formation, leading to an acetylation of both amino and hydroxyl groups present in chitosan. The final result was a colorless, transparent, and rigid gel composed of highly acetylated polymer chains, showing a degree of substitution of 2.36 per monosaccharide unit. Molecular aggregation between acetylated polysaccharide chains was proposed as gelation mechanism.

Some years later, Moore and Roberts gave insights into such gelling system investigating reaction variables such as the concentration of chitosan, that of anhydride, and the effect of temperature [25]. They concluded that an increase of temperature, anhydride and polymer concentrations speeded up the gelation process. Furthermore, they observed syneresis (about 50% of water loss) in the first 21 h after gelation. In a concomitant document the same authors attempted at elucidating the mechanism of gelation: this seemed to take place through the formation of crosslinks, likely ensured by hydrophobic interactions between A residues, leading to the formation of micellar junction points [26]. Moore and Roberts observed that gelation occurred upon reaching approximately 70% of formation of type A sugars, thus switching the nature of chitosan from a polyelectrolyte to a hydrophobic polysaccharide characterized by very low solubility in aqueous solutions. The final result was a collapse of the highly extended polymer to more compact entangled chains.

The same gelling system was further studied by Domard and co-workers [27]. They confirmed most of the evidences reported by Moore and Roberts, identifying some critical parameters allowing for gel formation. For instance, a minimum molar ratio *R* between the anhydride over D sugars of 1.3—yielding chitosans with F_A_ around 0.8—was found necessary for the complete gelation. Moreover, it seemed that a threshold degree of polymerization was necessary for the formation of macroscopic gels, since 30 > $\bar{DP_{w}}$ > 280 allowed only the formation of micro-gels. Beyond a degree of polymerization of 280, macroscopic networks formed. The influence of the nature of co-solvents (alcohols) was also investigated. In fact, the presence of co-solvents such as methanol was found to be fundamental in order to prevent *O*-acetylation, [28] and to generally decrease the dielectric constant of the media, thus fostering the *N*-acetylation process. Domard et al. demonstrated that both ethanol and 1,2-propanediol were suitable for gel formation, albeit showing different kinetics. Indeed, the gelation process was more rapid using the former co-solvent, likely due to its low viscosity with respect to the latter alcohol. Apart from kinetics aspects, it seemed that the two solvents showed no difference upon gelation, suggesting that the alcohol was necessary solely to avoid any side reaction [27]. The resulting gels were studied in a further contribution in terms of syneresis and swelling, pointing at the pivotal role played by the final acetylation degree, since weak syneresis and swelling phenomena were observed up to F_A_ = 0.88. Above this value, the weight loss increased with F_A_ [29]. Swelling experiments demonstrated that the solvent exchange between gels and surrounding environment was not fully reversible due to the formation of new junctions during the de-swelling step. Overall, given the (weak) polyelectrolyte character of highly acetylated chitosans used in this study, the syneresis was found to depend on pH and the ionic strength of the media.

The same authors proposed the application of such gelling system as injectable material for periodontal surgery purposes [30]. More in detail, they fine-tuned the optimal conditions of injection by varying different parameters, such as the molar ratio *R*, the temperature, and the concentration of chitosan. The optimum experimental conditions limited the syneresis, improved the mechanical properties of the gel, and determined a viscosity suitable for the injection.

**Figure 2:**
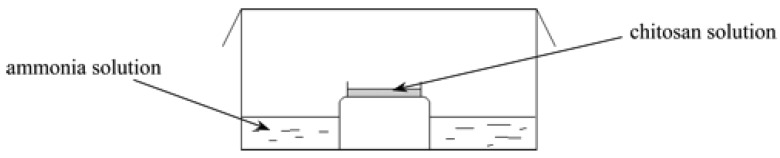
Experimental setup for the gelation of chitosan solutions by using gaseous ammonia atmosphere. Adapted with permission from [31], Copyright 2005 American Chemical Society.

Physical chitosan gels were also formed without the use of any external crosslinker in the presence of an aqueous ammonia solution as described in [31] (Figure 2).

In order to allow for gelation, three key conditions had to be met: (i) the initial concentration of solutions had to be over the critical concentration of chain entanglements, C*; (ii) the hydrophobic/hydrophilic balance had to achieve a critical value; and (iii) an interphase, corresponding to a bidimensional sol-gel transition, had to be created uniformly. The latter condition was achieved by the use of gaseous alkaline ammonia instead of an alkaline solution, since a heterogeneous gelation was noticed when chitosan was dialyzed against an aqueous ammonia solution. In these experimental conditions, the NH_3_ progressively neutralized the (weaker) primary amine functions of chitosan starting from the surface of solution to the bottom of the reactor, thereby promoting a decrease of the apparent charge density of chitosan chains. Upon exceeding a critical value, the transition sol-gel was demonstrated, wherein hydrophobic interactions and hydrogen bonds both cooperate to the formation of the network. The authors claimed that gelation kinetics could be speeded up upon increasing the F_A_ of chitosan samples (meaning that type A sugars are pivotal for the setting up of hydrophobic interactions) and the polymer concentration. The key role played by the hydrophobic interactions in the formation of macroscopic gels was also proved by assessing the mechanical properties of this set of materials. Specifically, the authors found that the equilibrium storage modulus, G_e_, which corresponds to the value of elastic modulus at the plateau for low frequencies, increased upon increasing F_A_, stemming from a higher number of junctions per unit volume. Furthermore, it was found that the influence of the polymer concentration, C_p_, on G_e_ was more important than that of F_A_, since G_e_ scaled with C_p_ according to the following power law: $G_{e}\propto C_{p}^{2.3}$, suggesting that the presence of more entangled polymer chains positively affected the formation of a higher amount of junctions per unit volume than the number of type A sugars in the sample. Overall, the authors identified the optimum experimental conditions for gel synthesis, which resulted to have an F_A_ value around 0.40, and a final polymer concentration close to 1.5% *w*/*w*.

Recently Fiamingo et al. exploited gaseous ammonia for gelling chitosan solutions at different polymer concentration. Resulting gels were used in vivo to evaluate the potential regeneration of infarcted myocardium, showing that such materials were well incorporated onto the epicardial surface of the heart, with a general lack of toxicity [32]. In parallel, physical chitosan gels were synthesized and subsequently fragmented, in order to obtain an injectable suspension of micro-gels in a range of dimensions around 20 µm [33]. This type of material was devised for spinal cord injury restoration and axon regeneration. Experimental data using a spinal cord injury model in rats demonstrated that the implantation of such micro-gels allowed the repair, to some extent, of spinal tissue without being combined with additional drugs.

The formation of chitosan macro-gels was also investigated by the same research group using hydroalcoholic media [34,35]. Chitosans at different F_A_ were solubilized using acetic acid or hydrochloric acid as solvent and alcohols were added in equal amount. The resulting mixtures were continuously stirred and water was allowed to evaporate up to the formation of tridimensional networks. In this approach the hydrophilic/hydrophobic balance is shifted toward the latter, thus promoting the formation of hydrophobic interactions and hydrogen bonds among chains. The authors demonstrated that water completely evaporated within approximately 1000 min, and the resulting mixtures were composed only by chitosan, alcohol, and residual amounts of acids used for the polymer solubilization. One key aspect to consider concerns the choice of the alcohol, whose boiling point must be higher with respect to water. The role of alcohol was to reduce the dielectric constant of the medium and possibly to participate in the formation of hydrophobic interactions between chitosan chains. At the end of the process, resulting gels were neutralized using sodium hydroxide and then washed extensively in order to eliminate any trace of alcohol. Hence, the final material was composed only by chitosan and water. Critical experimental variables for such gelation mechanism were: (i) the apparent charge density of chitosan; (ii) the dielectric constant of the solvent; (iii) F_A_; (iv) the temperature. The kinetics of gelation was also investigated by rheometry, [36] pointing at a key role of F_A_ and polymer concentration in shortening the gelation time; furthermore, highly acetylated chitosans showed higher mechanical properties.

The gelation of chitosan using hydroalcoholic media has been exploited for the fabrication of bio-inspired bilayer physical gels for the treatment of full-thickness burn injuries, showing that such chitosan-based materials were well tolerated in vivo, promoting tissue regeneration [37]. A controlled interruption of gelation has been achieved by simple imbibition of non-neutralized alcohol gels in sodium hydroxide, [38] thus generating an onion-like multi-membrane. It was found that the number of membranes composing the final gel could be easily tuned by varying the neutralization steps. Such multi-membrane materials showed pores in a range of tens of nanometers, which do not allow cell infiltration [39]. Anyway, this kind of materials proved to support the aggregation of chondrocytes without losing their phenotype when cells were loaded directly within the gels. Chitosan hydroalcoholic solutions also allowed for the formation of macroscopic gels when neutralized using NaOH or NH_4_OH [40]. Indeed, Rami et al. demonstrated the formation of physical chitosan gels characterized by different stiffness (Young’s modulus in the range 1–31 kPa, depending on F_A_). This set of materials behaved as suitable substrates for cellular anchoring, albeit no infiltration throughout the gels was noticed in vitro. It was found that the cell morphology and spreading depended on the gel stiffness: specifically, highly deacetylated chitosans allowed for the formation of stiffer gels, which proved to better foster cell spreading.

## 5. Ionic Chitosan Macro-Gels

When a polycation such as chitosan is fully charged, meaning that it is well below its pK_a_, and multivalent anions or negatively-charged macromolecules are added to the solution, two phenomena are essentially expected to occur: (i) if the concentration of chitosan, C_P_, is below or close to that of chain entanglement, C* (calculated as the inverse of the intrinsic viscosity 1[η]) [34,41] an ordered liquid-liquid phase separation—simple or complex coacervation based on the number of macromolecules involved in the process—will manifest only in the case of very precise values of the [crosslinker]/[chitosan]_r.u._ molar ratio; (ii) on the contrary, for C_P_ > C*, the system will evolve into irregular macro-aggregates that will precipitate. If the concentration of chitosan is quite larger than C*, whatever the crosslinker amount, inhomogeneous gelation and, often, precipitation phenomena will occur. Such phenomena are mostly governed by the very fast self-assembling of the polyeletrolyte when exposed to anionic crosslinkers. Hence, proper methodologies are required to form homogeneous ionic chitosan macrogels.

**Figure 3:**
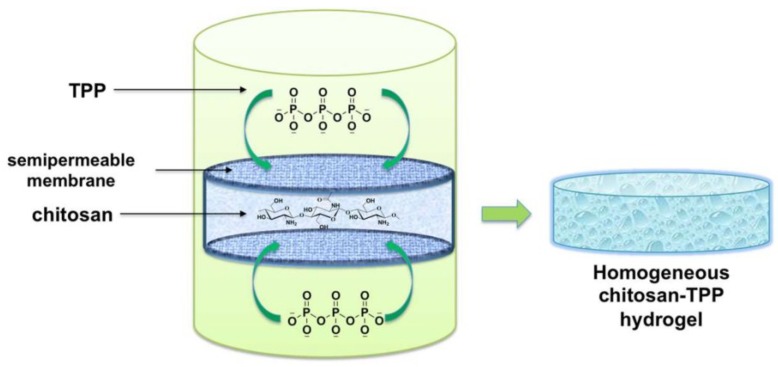
Experimental setup for developing chitosan-tripolyphosphate (TPP) macroscopic gels via external gelation. Adapted with permission from [42], Copyright 2014 American Chemical Society.

Sacco et al. reported the fabrication of cylindrical chitosan gels through a controlled external gelation using tripolyphosphate (TPP) as crosslinker. TPP is a multivalent anion, which shows from one to five negative charges depending on pH. A typical gelation requires that chitosan is acid solubilized in order to protonate all D sugars. The resulting chitosan solution was casted into a mold, and subsequently placed in a gelling bath containing TPP (Figure 3) [42].

This approach avoids the instantaneous gelation of chitosan and allows for a uniform distribution of the crosslinker throughout the solution. The effect of experimental variables, such as polymer and TPP concentration and of the presence of the supporting monovalent salt NaCl were explored to find the suitable conditions to form homogeneous cylindrical gels. The mechanical properties were found to vary simply by changing the concentration of chitosan or that of the crosslinker. The multivalent anion pyrophosphate (PPi) was also investigated for the fabrication of such gels [43]. For both the crosslinker types, i.e., TPP and PPi, it was hypothesized that the macroscopic gelation occurred through three consecutive steps, the first two common with what is known for micro-gel formation [23]: (i) the formation of small primary complexes; (ii) the subsequent aggregation into larger micro-gels of higher-order; and (iii) the aggregation of micro-gels via both electrostatic and van der Waals interactions among chains. Contrary to the chitosan-TPP gelling system, it was found that PPi allowed for the formation of inhomogeneous gels characterized by lower mechanical properties. Very recently it was demonstrated that the formation and mechanical properties of this set of materials could be strongly affected by intrinsic polymer features, such as its molecular weight, and its chemical composition, as F_A_ [10]. Specifically, it was found that the complete wall-to-wall gelation of chitosan required a minimum average of 152 sugars per chain; the gel stiffness increased with increasing molecular weight while the elasticity was almost independent of molecular weight. By varying the F_A_, it was possible to switch from very stiff and brittle gels to weaker but much more elastic ones. This kind of materials were studied for biomedical purposes as such, or freeze-dried in order to obtain soft and pliable membranes [42,44,45]. In the first case, chitosan gels did not show any cytotoxicity when put in contact with mammalian cells. The ability to host cells was verified by means of confocal microscopy, pointing at the pivotal role played by such matrices in cellular anchoring. Furthermore, chitosan gels were found to behave as suitable depots of macromolecules, since the release kinetics can be modulated by changing the crosslinker type or the molecular weight of payload [45]. Chitosan gels were freeze-dried to obtain pliable membranes as such, or implemented with silver nanoparticles. The latter material showed very interesting antibacterial properties without being cytotoxic toward mammalian cells, thus being a suitable candidate for the treatment of non-healing wounds [44].

A rapid ionic gelation of chitosan using a 6-phosphogluconic trisodium salt (6-PG-Na^+^) was recently reported [46]. The formation of a macroscopic gel was demonstrated by rheometry. From the biological point of view, such gels did not cause toxicity and dermal irritation, with potential application as vehicle for topical administration or as wound dressing.

**Figure 4:**
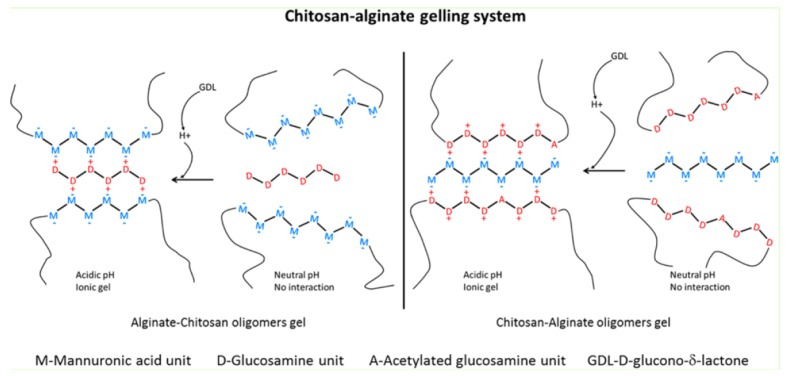
Gelling concept for synthetizing alginate-chitosan oligomers (**left**) and chitosan-alginate oligomers (**right**) gels via controlled internal gelation. Adapted with permission from [14], Copyright 2013 American Chemical Society.

Vårum and co-workers reported on the fabrication of macroscopic ionic chitosan gels using mannuronan oligomers as crosslinkers (Figure 4) [14].

This gelling system is based on two main steps: the first one requires that a medium acetylated chitosan (F_A_ = 0.40) is mixed with mannuronan oligomers at a pH around 7.5. Chitosans with F_A_ ≥ 0.40 are well known to be soluble at approximately neutral pH and above, [15] showing a small charge density. This allows such chitosans to be mixed with polyanions without phase separation. The second step requires the addition of a proton donor such as glucono-δ-lactone (GDL) in order to slowly decrease the pH of the mixture. It follows that chitosan progressively gains positive charges due to the protonation of amino groups at C2 position. Hence, electrostatic interactions with mannuronan oligomers take place in a range of pH 4–6. The final material is an ionic gel with peculiar mechanical properties. In the same contribution, the authors demonstrated that the present gelling concept worked also inverting the electrical nature of the components, inasmuch an alginate (anion) gel was obtained using chitosan (cation) oligomers (Figure 4).

Ionic chitosan gels could be obtained also using metallic anion based on Mo(VI) [47]. In the case reported by Draget et al. a dispersion of MoO_3_ is added to a chitosan solution and the sol-gel transition was verified by rheometry. The gelation mechanism is based on the gradual ionic interaction under acidic conditions between molybdate polyoxyanion (Mo_7_O_24_^6−^) and D residues on chitosan. Resulting gels showed lower mechanical properties if compared with Ca^2+^-alginate gels produced via internal gelation using similar experimental conditions, due to the different binding mode of Mo_7_O_24_^6−^ to chitosan with respect to Ca^2+^ in alginate. Finally, the authors demonstrated that Mo containing chitosan gels swelled/deswelled depending on the ionic strength of the incubation medium.

## 6. Ionic Chitosan Micro- and Nano-Gels

Chitosan forms polyelectrolyte complexes with negatively charged small molecules or macromolecules if the concentration of polymer is below or close to C*. Different polyanions have been extensively investigated to form micro- and nano-gels with chitosan, including inorganic ions such as TPP, polysaccharides as hyaluronan or alginate and synthetic polymers [48,49]. The ionotropic micro- or nano-gelation of chitosan usually requires a single injection or a dropwise titration of the polyanion into the chitosan solution at room temperature until a turbid system is obtained [50,51], thus indicating the onset of coacervation. The final outcome is a phase separation where a more viscous polymer rich phase—the “coacervate”—is noticed [52]. There are two ways to define coacervation phenomena. The “simple” coacervation refers to those systems composed by only one macromolecule, while “complex” coacervation deals with systems containing at least two—oppositely-charged—polyelectrolytes.

### 6.1. Simple Coacervation

The first evidence of the possibility to form nano-gels based on chitosan was reported by Calvo and co-workers in 1997 [50], with the use of TPP as the crosslinker. The authors evaluated the influence of chitosan and TPP concentrations on the physical-chemical properties of resulting nano-gels. Three different results were observed: (i) solutions at high chitosan concentration and low TPP amounts; (ii) insoluble aggregates at low chitosan concentration; and (iii) nano-gels at intermediate chitosan and TPP concentrations.

**Figure 5:**
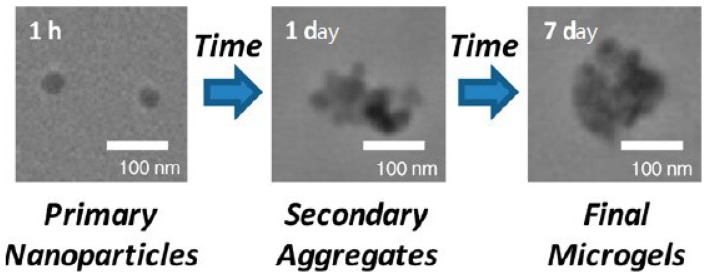
Formation of chitosan-tripolyphosphate (TPP) microgels via salt-assisted mechanisms. The final micro-gel is formed by a controlled aggregation of primary nanoparticles into larger coacervates. Adapted with permission from [23], Copyright 2012 American Chemical Society.

Lapitsky and co-workers extensively investigated the formation mechanism of chitosan/TPP micro-gels and their properties by tuning different parameters such as F_A_ and molecular weight of chitosan, pH, ionic strength and concentrations of polymer and crosslinker [23,53,54,55,56]. The kinetics of gelation was dramatically slowed down simply tuning the TPP and monovalent salt (NaCl) amounts [23]. Micro- and nano-gel formation rates resulted to be extremely sensitive to NaCl and TPP concentrations, and the formation process occurred in two stages as already discussed (Figure 5). The same authors also proved that large amounts of NaCl (e.g., 150 mM) enhanced the colloidal stability of chitosan/TPP micro-gels during their formation. In those experimental conditions, chitosan-TPP binding was weakened, so the bridging of the newly-formed micro-gels was inhibited, leading to narrow size distributions. Conversely, at high ionic strengths (e.g., 500 mM) the chitosan-TPP binding was weakened to the point that micro-gels did not form anymore. The suitability for biomedical applications of the chitosan/TPP micro-gel system is strictly influenced by its stability to aggregation and dissolution. Huang et al. investigated the effects of chitosan F_A_ and particle concentration at physiological pH and ionic strength [55,56]; the aggregation rate increased with increasing pH and decreased with increasing F_A_.

Chitosan-based nano-gels usually tend to aggregate after lyophilization and spray-drying [57]. Rampino et al. investigated the possibility to improve nanoparticle stability after drying in the presence of cryoprotectants such as trehalose, PEG and mannitol [58]. Trehalose was the best performing agent, and resulting formulations resulted biocompatible according to in vivo tests using chick embryos.

Chitosan/TPP micro- and nano-gels were widely investigated as systems for drug, protein, and gene delivery [59,60,61,62,63,64,65,66]. Bovine serum albumin (BSA) was frequently used as a model payload [50,59,67]. These micro- and nano-gels proved greater protein loading capacity and provided a continuous release of the payload up to one week [50]. Cai and Lapitsky systematically analyzed protein uptake performance as a function of micro- and nano-gel yields [67]. The protein association efficiencies were shown to scale almost linearly with the particle yield—which increased with the TPP and protein to chitosan ratios—until complete chitosan aggregation into particles. Upon increasing molecular weight of chitosan, BSA encapsulation efficiency was enhanced and its total release at physiological pH and ionic strength was reduced. By lowering the F_A_ of chitosan, the encapsulation efficiency was enhanced and the release rate was slowed down [68].

Due to the muco-adhesive properties of chitosan [69], nano-gels were widely studied as potential nano-carries of bioactive molecules in mucous-rich tissues such as ocular and nasal ones [62,63]. Cyclosporin A (CyA) was chosen as a model compound because of its potential usefulness for the treatment of local diseases; in vivo experiments showed that, following topical injection of CyA-loaded chitosan nano-gels, it was possible to achieve therapeutic concentrations in external ocular tissues during at least 48 h while maintaining negligible CyA levels in undesired zones (inner ocular structures, blood and plasma) [62]. Chitosan/TPP nano-gels were also able to encapsulate extremely hydrophobic drugs such as anti-hypertensives. Such systems encapsulated these drugs with high efficiency and were able to release them in a controlled way [70]. This type of nano-gels was even suitable for the encapsulation of anticancer agents such as doxorubicin [71].

### 6.2. Complex Coacervation

Complex coacervation of chitosan can be achieved through electrostatic interactions with negatively charged polysaccharides mixed in the right proportions. The most widely used anionic polysaccharides are hyaluronan, alginate, dextran sulfate, and carrageenan [49]. Such polyelectrolyte complexes are commonly used as drug delivery systems in very different fields of application, spanning from the delivery of genes to that of proteins [72].

#### 6.2.1. Hyaluronic Acid

One of the most interesting polyelectrolyte chitosan-based complex is that formed with hyaluronic acid (hyaluronan, HA) [73]. Chitosan-hyaluronan nano-gels are typically obtained by dropping hyaluronan into a chitosan solution [51,74]. Conceivably, the driving forces for nano-gels self-assembling are the gain of entropy due to the release of water molecules and counterions from both polysaccharides together with electrostatic interactions between the two oppositely-charged polyelectrolytes [75].

Very little is known about the influence of F_A_ on the stability of resulting nano-gels and its biological implications, since most authors used commercial chitosans with very similar physical-chemical properties, typically F_A_ < 0.2 and medium/high molecular weight. Delair and co-authors reported limited stability of polyelectrolyte complexes based on chitosan and hyaluronan [76]. The authors claimed that the stability of such coacervates, prepared in medium with ionic strength and pH close to physiological conditions, could be reached adding metallic ions (Zn^2+^).

Some of the authors of the present review evaluated the role played by F_A_ in the stability and biological activity of chitosan-hyaluronan nanogels [77]. Systems made with low molecular weight chitosans were devised for the encapsulation of anti-inflammatory molecules and were able to modulate several neutrophil functions [51]. Specifically, such nano-gels were developed as controlled release carriers in order to provide a long-lasting supply of payload, resulting in a limited production of neutrophil reactive oxygen species (ROS). Muco-adhesive properties of nano-gels based on chitosan and hyaluronan can be exploited for gene delivery as well as transmucosal delivery of drugs and proteins. CH/HA nano-gels were able to encapsulate heparin and were promising nano-carriers for pulmonary delivery applications [78]. They were also suitable for the encapsulation of proteins such as the growth factors VEGF and PDGF-BB [79]. Nano-gels also encapsulated plasmids with high efficiency and were promising carriers for ocular gene therapy [75], reaching transfection levels up to 25% in cell models [80].

Hyaluronic acid is of particular interest due to its ability to interact with membrane receptor CD44, [81] known being overexpressed in certain cancers, such as those affecting the breast [82] and liver [83], thereby cueing antineoplastic drugs for a selective targeting. Deng and co-authors developed chitosan-hyaluronan nano-gels suitable to overcome drug resistance in breast cancer [84]. An antineoplastic drug, doxorubicin, and miR-34a, a potent endogenous tumor suppressive molecule, were co-encapsulated in nano-gels. In vitro and in vivo experiments showed that nano-gels enhanced anti-tumor effects of doxorubicin and miR-34a, thus resulting a promising therapeutic strategy for enhancing anti-tumor therapy. Lallana et al. studied the role of RNA avidity for chitosan on the transfection efficiency by using a small library of chitosans (variable molecular weight and F_A_) in CD44-expressing cells [85]. Chitosan with high F_A_ showed limited stability, were poorly internalized and, therefore, were not effective in RNA delivery. The best transfecting particles were those with lower F_A_. Tirelli and co-workers deeply investigated the possibility to use a hyaluronan coating on chitosan-TPP nano-gels [81,86,87,88,89]. They reported that hyaluronic acid coating reduced the immunogenity and toxicity of nano-gels [87,88,89]. Almalik et al. provided evidence that CD44-mediated endocytosis was responsible for the uptake of materials where HA was used as the coating agent [81,90]. The same author evaluated the influence of chitosan molecular weight on physical-chemical properties of the nano-gels and DNA loading [86]. Molecular weight did not influence DNA loading capacity; conversely, it influenced the physical-chemical properties of nano-gels. Specifically, higher molecular weight chitosan increased porosity (i.e., decreased the crosslink density), also causing larger dimensional changes upon varying the osmotic pressure or after drying. HA penetrated deeply into the more porous nano-gels composed of high molecular weight chitosan, whereas formed a corona in lower molecular weight chitosan nano-gels [86].

#### 6.2.2. Alginate

Alginate is another widely used polysaccharide to form chitosan-based nano-gels [49]. Stokke and co-workers investigated the possibility to synthetize coacervates using polymers with different physical/chemical composition and by varying the modality of mixing of the two polysaccharides [91]. The mixing order influenced the size of nano-gels, whereas the net charge ratio between chitosan and alginate and the molecular weight of both polymers affected both the particle size and surface charge. Abreu and collaborators investigated the influence of alginate content, chitosan molecular weight and micro-gel preparation method on the reaction yield, particle size, and swelling degree [92]. The main parameter affecting the reaction yield resulted the chitosan molecular weight. By tuning the alginate content it was possible to vary the charge density and the swelling degree of the micro-gels. The same authors provided an equation aimed at correlating different parameters. Hence, it was possible to predict the desired features for a controlled drug release of payloads by properly tuning some properties of the initial system.

#### 6.2.3. Dextran Sulfate

Costalat et al. developed reversible nano-gels based on chitosan and dextran sulfate [93]. Nano-gels formed and dissolved simply by tuning the ionic strength of the system via dialysis. In the presence of an excess of polycation, colloidal gels were exclusively obtained. At low salt concentrations, the spontaneous gelation provided macro-gels with different homogeneity [94]. Weber and collaborators investigated the possibility to use nano-gels based on chitosan and dextran sulfate as potential delivery systems for an antigenic protein (p. 24, the capsid protein of HIV-1), useful as vaccine carriers [95]. They tuned experimental conditions, molecular mass and F_A_ in order to retain the colloidal properties of the carrier in physiological conditions. After subcutaneous injection in mice, a specific immune response was observed and both the cellular and the humoral responses were stimulated.

#### 6.2.4. Carrageenan

Rodrigues et al. studied the possibility to form nano-gels based on chitosan and carrageenan [96]. The further addition of TPP reduced the dimensions and improved the stability of the nano-gels. The authors microencapsulated BSA by spray-drying and resulting formulations showed good biocompatibility toward different cell lines. Such nano-gels were shown to be promising as protein carriers for pulmonary and nasal transmucosal delivery [97].

## 7. Thermosensitive Gels

Thermo-sensitive gelling systems consist of stable, low viscosity, aqueous solutions that turn into a gel state upon heating. The sol-gel transition usually occurs at neutral pH and takes a few minutes [98,99]. The first evidence of the possibility to synthesize thermosensitive gels based on chitosan was reported in 2000 by Chenite and collaborators [100]. Thermo-sensitive gelling systems were devised by combining chitosan and polyol-phosphates, polyol-bearing molecules, or inorganic phosphate molecules. At first, β-glycerophosphate (β-GP) was used as gelling agent, [100] then the attention moved also toward other polyol-phosphates such as glucose-1-phosphate (G1-P) and glucose-6-phosphate (G6-P) [101]. As polyol molecules, 1,3-propanediol, 1,2-propanediol, mannitol, trehalose, and mannitol were used as well. As inorganic gelling agents, hydrogen phosphate salts—ammonium, sodium or potassium—were also used [99].

**Figure 6:**
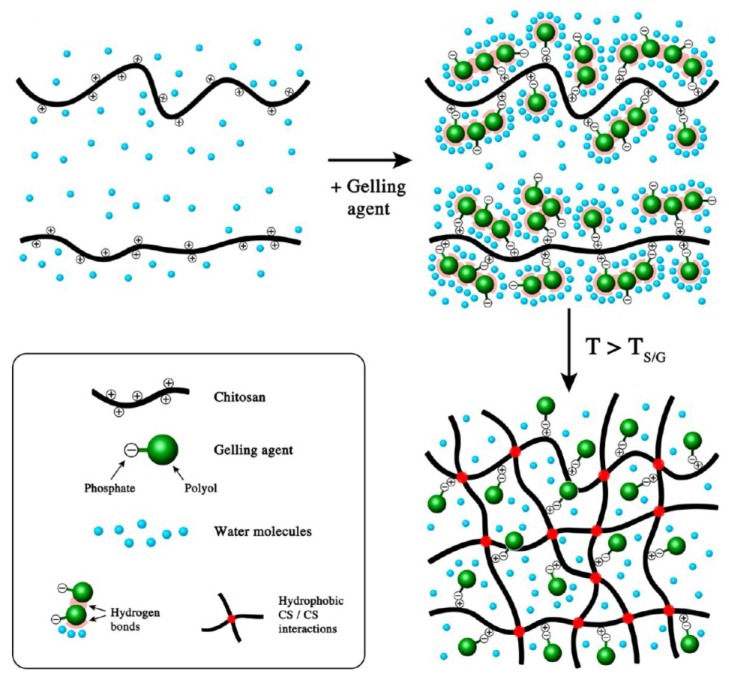
Gelling mechanism of chitosan polyol-phosphates solutions. Adapted with permission from [101], Copyright 2013 American Chemical Society.

The gelling mechanism of chitosan in the presence of β-GP was elucidated by Lavertu and co-workers. They proposed that precipitation and gelation of chitosan derived from heat-induced transfer of protons from chitosan to β-GP [102]. This phenomenon would induce charge neutralization in chitosan, facilitating hydrophobic interactions between polymer chains and allowing attractive inter-chain forces to form a physical gel (Figure 6). By using β-GP as a crosslinker, the gelation temperature, depending on the amount of β-GP, ranged from 15 to 85 °C. The resulting fraction of protonated glucosamine monomer was temperature independent but increased with decreasing chitosan F_A_ and with increasing salt content. Supper et al. further investigated the mechanism of the gelation process, comparing different systems based on chitosan and various polyol-phosphates, such as β-glycerophosphate, glucose-1-phosphate and glucose-6-phosphate, varying in the chemical structure of the polyol parts [101]. Macroscopic gelling behavior of the solutions, such as transition temperature, gelation time, and gel strength, indicated that increasing the size of the polyol part prevented the interactions between the chitosan chains, strongly influencing the gelling process.

Injectable thermo-sensitive gels have shown great potential in biomedical applications, including local drug delivery and tissue engineering [103,104]. Nevertheless, some authors reported problems about storage stability, both at room temperature, as well as at refrigerated conditions [98,105]. Neither lyophilization was able to retain the viscoelastic properties of the chitosan-based thermogelling solutions [106]. To improve the stability, additional polyol-phosphates, such as glucose-1-phosphate (G1-P) and glucose-6-phosphate (G6-P) have been investigated [101]. By using G1-P it was possible to improve the stability of formulations. Resulting systems were found stable for at least nine months at 2–8 °C, at variance with what was observed in the case of β-GP (one month) [98]. Furthermore, the solution was easy to inject and showed a sustained release for days to weeks for hydrophilic model compounds, thus demonstrating that the chitosan/G1-P system could be suitable for the prolonged delivery of drugs. Supper and co-workers also evaluated the influence of the molecular weight and concentration of polymer, as well as that of G1-P on thermogelling potential [98]. The decrement of chitosan molecular weight and its concentration resulted in a significant decrease of the gelation time, in agreement with previous reports based on β-GP [107]. The same effect was achieved by increasing the G1-P concentration, in agreement with previous reports on chitosan β-GP systems [98,108,109]. The sol/gel transition took place at around body temperature and was not fully thermo-reversible, confirming the potential of chitosan/G1-P solutions as an injectable ready-to-use forming gel in situ [98]. An alternative approach for improving the storage stability deals with the addiction of stabilizing agents. When trehalose or mannitol were used, the re-hydrated sample displayed thermosetting properties, thereby retaining injectable properties [106].

Giving the peculiar properties of this set of materials, Chenite and co-workers undertook biological tests: specifically, they injected such formulations, which gelled upon implantation in vivo; all implants were intimately integrated within the subdermal fibrous membranes. Formulations were used also as an encapsulating matrix of chondrocytes for tissue engineering applications [100].

These thermogelling solutions were widely studied for the development of parenteral drug delivery systems, which enabled a sustained release of potentially encapsulated active substances [101]. In order to achieve a prolonged release of low molecular weight payloads, drug-loaded carrier particles were also incorporated in the thermogels [99]. In case of a further strengthening of the network was needed, an additional polymer and/or additives were used. Such biodegradable drug delivery systems were suitable as in situ forming depots (ISFD) after subcutaneous administration and were exploited in different applications. Nevertheless, some authors reported that an inflammatory reaction occurred in the tissue surrounding the gel [98,110].

Thermosensitive gels based on chitosan and β-GP were studied as materials for cartilage and bone regeneration. Hoemann and co-workers described the utility of such materials as arthroscopically-injectable vehicles for cell-assisted cartilage repair [111]. They developed a cytocompatible chitosan solution that was space-filling, gelled within minutes, and adhered to cartilage and bone. The in situ-gelling chitosan solution was able to support in vitro and in vivo accumulation of cartilage matrix by primary chondrocytes, while persisting in osteochondral defects at least one week. Huang and co-workers developed a biphasic scaffold platform able to retain cells homogeneously [112]. This biphasic scaffold platform was functionalized with an affinity peptide targeting bone marrow stem cells (BMSC), to provide BMSC-specific homing both in vitro and in vivo. The functional biomaterial stimulated stem cell proliferation and chondrogenic differentiation in vitro. After in vivo implantation, compared with routine surgery or control scaffolds, the functional biomaterials induced higher cartilage repair without complications. This functional biphasic scaffold could provide a biomaterial framework for one-step tissue engineering strategy by homing endogenous cells to stimulate tissue regeneration. BMSC were also successfully encapsulated in gels supplemented with collagen [113]. The presence of chitosan increased the alkaline phosphatase activity and calcium deposition in osteogenic medium. These composite materials demonstrated potential as matrices for cell encapsulation and payload delivery, or as in situ gel-forming materials for bone repair.

Some authors reported issues about the inadequate mechanical strength of thermoresponsive gels based on chitosan for osteochondral repair [99,114]. To address such flaws, Huang and co-workers constructed a solid-supported thermogel comprising of a chitosan gel system and demineralized bone matrix [114]. Compared with either the simple gel or the demineralized matrix, the hybrid biomaterial showed superior porosity, equilibrium swelling and degradation rate. The hybrid scaffolds exhibited an increased mechanical strength. BMSCs maintained viability above 90% and the cell retention of the hybrid scaffolds was more efficient and uniform than other materials. Matrix production and chondrogenic differentiation of BMSC in the hybrid scaffolds were superior to its precursors. These experimental evidences indicated that that solid-supported thermogel could be an attractive biomaterial framework for cartilage tissue engineering [114]. An alternative approach to improve mechanical properties was aimed at including nano-crystals of β-tricalcium phosphate, the latter able to interact via physical interactions. The systems exhibited a gel-phase transition at body temperature, and a three-dimensional network with typical rheological properties of a strong gel. The presence of the inorganic phase provided a structure with physical/chemical composition akin to that of natural bone tissue. Overall, such formulations seemed suitable for hard tissue regeneration [115]. A composite scaffold was obtained by combining chitosan and demineralized bone matrices modified with the E7 affinity peptide, able to bind BMSC, for potential application in cartilage regeneration [116]. The composite scaffold presented an appropriate porosity suitable for supporting cell adhesion and proliferation, increased matrix production and improved chondrogenic differentiation ability in vitro. After implantation in vivo, much more cartilage-like structures than the control group were found. Such composite scaffold could be a promising option for repairing irregularly shaped cartilage defects.

The addiction of zinc (Zn) to thermosensitive gels allowed for enhancing antibacterial activity and promoted osteoblast differentiation, therefore having a potential as injectable in situ forming matrix for bone tissue engineering applications [117]. The antibacterial activity of the resulting formulations was successfully improved also by using nanocomposites materials based on silver and palladium. This set of materials proved osteogenic ability, displayed antibacterial activity and lack of toxicity. Hence, they are promising for dental surgery purposes [118].

Different approaches for obtaining thermosensitive gels based on chitosan were undertaken using inorganic phosphate hydrogen phosphate salts. Nair and co-workers reported that by adding different concentrations of ammonium hydrogen phosphate to chitosan solution it was possible to tune the gelling time from 5 min to 30 h at 37 °C [119]. These gels did not show any toxicity toward osteoblast-like cells. Moreover, they were suitable as stem cell and payload carrier. Ta and co-workers developed thermosensitive gels based on chitosan and inorganic orthophosphate [104]. These authors reported that monobasic and tribasic phosphate salts were not effective in inducing gelation of chitosan; conversely, dibasic phosphate salt such as dipotassium hydrogen orthophosphate was able to form gels. Gelation temperature and gelation time were tuned by varying chitosan and salt concentrations in the final formulation. Resulting systems were effective to release the entrapped macromolecules and were found to be cytocompatible. Li and co-workers detected two sol-gel transition temperatures using similar formulations, namely 4 and 37 °C. Strikingly, the formulation reverted to a solution around 30 °C [120]. By increasing the temperature up to 43 °C, a similar gel-sol transition, as well as a decrease in turbidity, was detected.

## 8. Interpenetrating Polymer Network (IPN)

**Figure 7:**
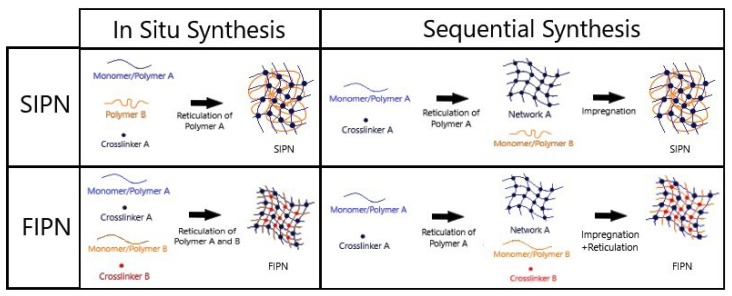
Classification of interpenetrating networks. SIPN: semi-interpenetrating networks (**upper row**); FIPN: full interpenetrating networks (**lower row**). In situ and sequential syntheses refer to the number of steps requiring for gel setting up.

Among physical gels, an interesting category is represented by the interpenetrating polymer networks (IPNs). According to the definition given by the IUPAC Gold Book, IPNs are networks comprising two or more polymers that are at least partially interlaced on a molecular scale, but not covalently bonded to each other and cannot be separated unless chemical bonds are broken. Unlike blends of heterogeneous polymers, IPNs show a network of chains held together by physical entanglements or weak interactions and, thus, are intimately mixed and mechanically interlocked [121]. In light of this feature, the main advantage of IPNs is to combine the best properties of single-network gels and the properties of a system in which the cooperation between chains makes it possible to achieve superior mechanical and biological features, as the sum of the contribution from both polymers. Hence, an IPN is an advanced multicomponent polymeric system in which it is possible to tune the properties by choosing the polymer combinations [122]. Both synthetic and natural macromolecules are used to such a purpose. Synthetic polymers are chosen to create medical devices because, in general, they show elevated mechanical strength, are cheap, easy to prepare, and can be chemically modified. In contrast, natural biopolymer gels are highly biocompatible, easily biodegradable, and can induce specific cellular responses. In spite of this, they often show lower mechanical strength [123]. In this context, IPNs can be made by both synthetic and natural polymers: the former would provide the right stiffness and support, while the latter would enhance cellular adhesion and stimulate growth and metabolism [124]. IPNs, as reported in [125], can be classified according to two basic criteria (Figure 7). The first classification is of morphological nature, as it is based on the final structure of the polymers that form the network; it divides them into full-interpenetrating networks (FIPNs), often defined just IPNs to simplify, and semi-interpenetrating networks (SIPNs).

FIPNs are defined as 3D systems of two (or more) polymers each of which is crosslinked separately [126], while SIPNs are composed of a single crosslinked polymer in which a second linear or branched polymer is added [122]. Generally, FIPNs are more stable and show higher mechanical strength than SIPNs; moreover, in FIPNs it is possible to better control their porosity by choosing the degree of crosslinking of both networks. The features of FIPNs find application in tissue engineering and in the design of cell scaffolds [124]. SIPNs show reduced stiffness but, in some cases, present a behavior that can be desirable for the use in biological systems. For instance, pH-dependent swelling, strength tuning by temperature or self-healing abilities were reported due to the bridging of cracks by diffusion of the non-crosslinked polymer chains while structural integrity was provided by the crosslinked chains [127]. Thanks to such features, SIPNs are ideal systems as drug delivery networks with controlled release [128].

The second classification criterion refers to the number of steps required for the synthesis and distinguishes between IPNs produced by sequential and in situ synthesis [125]. The most widely used approach is the sequential synthesis that consists in the formation of the first network by using a polymer (A) and a crosslinking agent in the first step; then, the network is immersed in a solution containing the second polymer (B) or its precursors and, through the swelling of the first network, the polymer/monomers B is embedded into the network A (impregnation step); when polymer B is crosslinked in a second moment, it becomes a FIPN. Instead, if polymer B is left uncrosslinked, the system remains a SIPN. This method provides the advantage of being easily modified and adapted to different gel systems, but it requires large amounts of the second polymer to reach an efficient swelling. It takes a long time (about 1–2 days) to complete the process and it is tailored for crosslinked polymers that hardly behave as self-healing gels. In contrast, more recently the in situ approach has gained much attention. It consists in mixing together, in the same reaction medium, both polymers and crosslinkers; in the case of FIPN, the formation of the two networks can occur simultaneously or manifest in two different moments. Nevertheless, there is a higher probability of cross-reactions between various components, thus it is important to choose the right crosslinking molecules. The main advantages of using the in situ approach are the speed of the process, which can reach completion in 1–2 h, the reduced number of passages that makes the whole procedure easier, and its high reproducibility [129].

The overall tendency is to design medical devices based on IPNs that can mimic the ECM, thus allowing and enhancing the adhesion and growth of cells on the substrate. Chitosan represents an ideal polymer for such a purpose. Chitosans can behave as “smart” stimuli-responsive polymers when are part of gel systems, they can respond to external factors such as temperature, electric current, solvent composition, pH, and ionic concentration. Chitosan-based IPNs can be prepared for different applications in the biomedical field, such as drug delivery, tissue engineering, and scaffolding [22].

### 8.1. Drug Delivery and Wound Dressing

Hernández et al. proposed a full-IPN as micro-gel for drug delivery based on poly-*N*-isopropylacrylamide (PNIPAM) coupled with chitosan and synthesized according to the in situ technique [130]. Such system showed thermo- and pH-responsive behavior, thereby allowing for a controlled drug delivery. NIPAM and chitosan were mixed together in acetic acid, then NIPAM and *N*-*N*′-methylenbisacrylamide (BIS) were added. Finally, tetramethylethylenediamine (TEMED) and ammonium persulfate (APS) were mixed and let the reaction occurring. Finally, gels were dialyzed and freeze-dried. The gel showed a high percentage of swelling at low pH because of the presence of chitosan, while the response to temperature was principally due to PNIPAM, which had a lower critical solution temperature (LCST) of 32 °C. Under these experimental conditions amide groups in hydrophilic segments of the polymer and water molecules showed strong interactions because of hydrogen bonds. These characteristics made the gel as an ideal system for the setting up of controlled drug delivery devices. Another example of how to exploit the pH sensitivity of this kind of systems comes from [123]. In this work it is described a SIPN formed between a derivative of chitosan (*N*-Succinyl chitosan, NSC) crosslinked with glutaraldehyde and non-crosslinked poly-acrylamide-co-acrylic acid (poly(AAm-*co*-AA)) embedded into the chitosan network. The gel was synthesized by the in situ approach and showed a porous structure thanks to the high hydrophilicity of NSC, while the embedded Poly(AAm-*co*-AA) created more homogeneous pores. The system greatly swelled at pH 7.4 because of the presence of the carboxylic groups that deprotonated into carboxylate ions, thus causing repulsion of the chains. It has been tested for the controlled release of 5-fluorouracil, an anti-tumor and anti-metabolite drug in order to increase the leakage of the drug in the colon rather than in the stomach after oral administration. Furthermore, the semi-IPN could be also considered for tissue engineering purposes thanks to the excellent interconnected porous network, which favors an efficient exchange of nutrient and gas. The idea of controlling the gel swelling by multi-stimuli response systems has been deeply described in [131]. In this paper it is reported that a semi-IPN composed by chitosan was embed in a crosslinked network of polyacrylamide (PAAm) with an in situ synthesis. The resulting gel showed a different swelling upon changing of pH, temperature, ionic strength, and electric field applied, allowing to control in multiple and, thus, more sophisticated ways the release of payloads entrapped in its mesh. The system was proposed for drug delivery and wound dressing. Another example of in situ synthesis has been proposed by Treenate et al. [128] with a semi-IPN formed by a network of alginate ionically crosslinked with CaCl_2_ and a polymer of hydroxyethylacryl chitosan (HC) in which the crosslinker was added after mixing of alginate and HC: in this way HC was homogeneously dispersed into the matrix of alginate. The system was stable at pH 1.2, and degraded at pH 7.4, making the network useful for a controlled drug release. The tensile strength and elongation of the gel films were 12.1 MPa and 162%, respectively, granting the system with the mechanical properties adequate for the development of wound dressings. A different way to interlace polymers is represented by UV treatment in the presence of a photoinitiator. As reported in [132], a SIPN was formed by a single reaction mixing a derivative of chitosan, the water-soluble *N*-carboxylethyl chitosan (CECS), and 2-hydroxyethyl methacrylate (HEMA). Both polymers were solubilized in water with a photoinitiator, then poured into a mold and finally irradiated by a UV light source. The system is suitable for transdermal drug delivery and wound dressing. Similar chitosan derivatives and partner polymers were used in applications somehow resembling the ones described above.

### 8.2. Tridimensional Cellular Scaffolds

An interesting application of IPNs is the design of 3D scaffolds for the growth of various cells, since they can mimic the cellular environment and improve cell viability and proliferation. Some authors worked on a SIPN composed of a chitosan-derivative (CML, with methacrylic and lactic acids sequentially grafted onto chitosan backbone) crosslinked in situ by UV light in the presence of an initiator to form the first network and then loaded with gelatin [133]. The gelatin addition reduced pores and the swelling ratio of gels, thus enhancing their strength. Physical-chemical features of the scaffold ensured the maintaining of morphology and phenotype of chondrocyte cells because the scaffold mimicked well the ECM. A very intriguing study is reported in [124] which is focused on the study of different type of cellular scaffolds made by chitosan and *N*,*N*-dimethylacrylamide (DMAAm) crosslinked with gamma-irradiation. In situ synthesis was performed for both SIPNs (only DMAAm crosslinked) and FIPNs. SIPNs showed a higher percentage of swelling compared to FIPN due to the higher crosslinking of DMAAm, while the degradation is higher in the case of FIPN as temperature increased. Moreover, the pore size was smaller and less dispersed in SIPN than in FIPN.

### 8.3. Bio-Electro Sensing and Soft Actuators

The capability of chitosan to respond to electric current variations has led to the development of biosensors and energy transducers that can find large applications in the field of electro-sensing and robotics. Kim et al. [134] reported that an IPN made of poly (vinyl alcohol)—PVA—and chitosan crosslinked by UV radiation by means of an in situ reaction could bend differently depending on the applied electric stimulus. The network showed a different swelling depending on salt concentration and temperature, due to the association and dissociation of hydrogen bonds between hydroxyl groups in PVA and the amino groups in chitosan. The high mechanical strength conferred by PVA and the good biocompatibility of chitosan made the system useful as a component for artificial organ, such as muscle-like contractile structures, sensors, switches, and electric current modulated drug-delivery systems. Few years later, the same authors developed another electric-responsive gel, i.e., a SIPN composed of chitosan and chemically crosslinked poly-hydroxyethyl methacrylate (PHEMA) [135]. This new kind of network was sensitive to electric current and showed various bending degrees depending on electric stimulus. Its electroresponsive behavior was also affected by electrolyte concentration of the external solution. The contribution of chitosan IPNs for the development of biosensor devices with regard not only to the direct response to various kind of stimuli, but also the entrapping of enzymes used to detect molecules of interest, as networks based on chitosan can mimic well the environment in which enzymes naturally operate. Zeng et al. describes a biosensor made of a carbon electrode covered with a film of semi-IPN composed of polyacrylamide (PAM) and chitosan [136]. The gel film could entrap hemoglobin in its native state and allowed the redox reaction for which the biosensor was built. This system represents an ideal model to study electron transfer by heme enzymes.

### 8.4. Tissue Engineering

Huang et al. developed a semi-IPN based on two types of crosslinking, both covalent and ionic [137]. The first network was obtained with covalent crosslinking of acrylamide and maleic acid and the simultaneous entanglement of a water-soluble carboxymethyl chitosan (CMC) in the mesh exploiting an in situ approach. Thereafter, following a sequential synthesis, the network was immersed into a solution of ferric ions, which further strengthened the gel and accommodated the strain by rapid reversible dissociation and association. The system showed a tensile stress of about 1.44 MPa and an antibacterial activity, thus suggesting it for many biomedical applications from wound dressing to gene/drug delivery and tissue engineering. The strategy of using molecules that naturally reside in the ECM to mimic an ideal situation for cell growing is often exploited in tissue engineering and the SIPN reported in [138] is a good example. The authors proposed an in situ synthesis for a semi-IPN composed of a network of crosslinked glycol-chitosan and type I and III collagen. The gel showed a nano-fibrillar porous structure and it was mechanically stable under continuous dynamic stimulation. These characteristics made the gel a good scaffold for cell viability, metabolism, adhesion, and migration, and could be proposed for uses in tissue engineering, as for the replacing of vocal folds or heart valves, which represent mechanically challenging tissues. An example of water absorbent material was reported by Wang et al. [126]. The gel studied was composed of a first network of gelatin crosslinked with chitosan; in a second moment, poly (vinylpyrrolidone), PVP, and its crosslinker were added to the first network following the sequential method. The innovation was represented by the use of microwave and ultrasonic techniques to obtain the crosslinking, allowing a very quick synthesis. The tensile strength was very high (approximately 90 MPa), making the system suitable for tissue engineering and biomedical applications, which would involve the need of high mechanical performance. Finally, an example of FIPN made by in situ synthesis and formed by poly (vinyl alcohol), chitosan, and poly (acrylic acid) is reported by Zhang et al. [139]. The network showed a high swell ratio, thus considering it as a potential modulator system in the biomedical field.

## 9. Chitosan Derivatives

Chitosan displays several interesting features, which have been discussed in the previous sections. However, poor solubility at neutral pH, especially for medium/high molecular weight chitosans with F_A_ < 0.4 and lack of specific biological signals are severe limitations to its large-scale use in the biotechnological field where direct contact with cells and/or tissues is foreseen. The derivatization of chitosan can overcome some of aforementioned flaws. The modification can be specific, involving the amino group (–NH_2_) at C_2_ position, or nonspecific, involving hydroxyl groups (–OH) at C_3_ and C_6_ positions [140]. The hydroxyl groups of chitosan have nearly equal reactivity. One of the most widely used methods to derivatize chitosan is the quaternization of the amino group to improve the antibacterial properties [141]. Another quite simple reaction refers to reductive amination. The reaction can be performed in aqueous solutions under mild conditions yielding randomly-distributed substituents along the chitosan chain [140].

In a paper by Diolosà et al. chitosan was modified with methacrylic acid (Chit-MA70) on 16% of the amino groups aiming at investigating its effect on the durability of adhesive interfaces to improve the clinical performance of dental restorations [142]. Chit-MA70 was blended into a primer of an “etch-and-rinse” experimental adhesive system and tested on human teeth. The presence of methacrylate moieties and of residual positive charges on the polysaccharide chain allowed Chit-MA70 to covalently bind to the restorative material and electrostatically interact with demineralized dentin. The Chit-MA70 containing an adhesive system showed values of the immediate bond strength (26.0 ± 8.7 MPa) comparable with the control adhesive system (25.5 ± 8.7 MPa). However, it was shown that upon performing thermo-mechanical cycling treatment of the dental restoration on human teeth, the adhesive with the methacrylate-modified chitosan, in variance with the control adhesive, did not show any decrease in the bond strength (28.4 ± 8.8 MPa). The modified chitosan was proposed by the Authors as a component of the “etch-and-rinse” adhesive system to efficiently improve the durability of dental restorations.

An interesting approach for the modification of chitosan backbone is based on the introduction of sugars as flanking groups. As an example, mannosylated chitosan was used for the specific recognition to antigen presenting cells such as B-cells, dendritic cells, and macrophages [143]. Other sugar derivatives of chitosan have been reported to show antioxidant activities [144] and enhanced release of nitric oxide, TNF-α, and IL-1 β in macrophages [145].

### Physical Gels Based on Chitosan Derivatives

The use of chemically-modified chitosans was widely exploited to enhance pH sensitivity or biodegradability of thermo-sensitive gels based on chitosan [99]. Interesting mechanical properties were detected by You and co-workers by using a gel based on a quaternized chitosan and poly (acrylic acid) [146]. The mechanical behavior of such gels was tuned from stiff and viscoelastic to soft and elastic by changing the poly (acrylic acid) content. Moreover, resulting gels displayed excellent shape-memory behavior due to the reversible properties of the ionic bonds and were endowed with self-recovery features. This peculiar behavior was attributed to the high charge density and solubility of quaternized chitosan, which enabled the formation of strong electrostatic interactions in the gels. Another interesting approach dealt with the modification of chitosan with two water-soluble thermosensitive polymers, namely poly (*N*-isopropylacrylamide), PNIPAm, and poly (*N*-vinylcaprolactam), PVCL [147]. Fang and co-workers grafted chitosan with poly (*N*-isopropylacrylamide) to obtain temperature-sensitive gels able to release in a controlled way both hydrophilic and lipophilic drugs in an in vitro drug release experiment [148]. In vitro cell culture experiments with chondrocytes and meniscus cells in the gels combined with hyaluronic acid showed beneficial effects on the cell phenotypic morphology, proliferation, and differentiation [149].

An alternative approach for fabricating thermosensitive gels based on chitosan derivatives was proposed by Tang and co-workers [150]. Carboxymethyl chitosan was combined with oxidized gellan gum, thus obtaining a gel with an almost physiological gelation temperature and able to significantly enhance the viability of encapsulated chondrocytes. The obtained formulation resulted to be a promising material for cartilage tissue engineering. Chitosan grafted with glycolic acid and phloretic acid were designed to obtain biodegradable injectable chitosan gels through enzymatic crosslinking with horseradish peroxidase (HRP) and H_2_O_2_ [151]. Short gelation times in the range of tens of seconds were detected culturing chondrocytes, revealing that cells were viable and retained their round shape. Such gels are promising as an artificial extracellular matrix for cartilage tissue engineering. An interesting use of chitosan and hyaluronic acid derivatives was proposed by Manna and co-workers [152]. Chitosan was grafted with adenine, whereas hyaluronic acid was modified with thymine and the self-assembly was obtained by means of hydrogen bonding between DNA base pairs substituted on the backbone of polymers. Alternated layers of each single polymer type were deposited on the surface, by obtaining a multilayer film, which did not show cytotoxicity.

## 10. Lactose-Modified Chitosan (CTL)

**Scheme 1:**
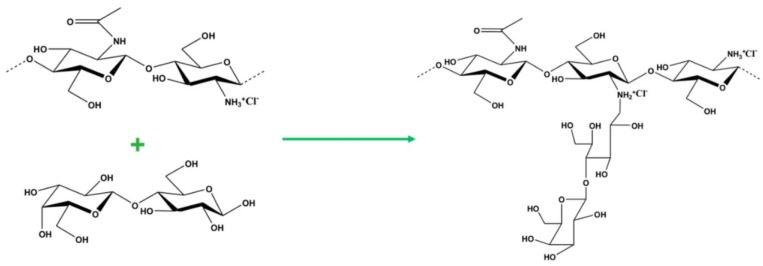
Synthesis of chitlac (CTL) through *N*-alkylation of chitosan in the presence of lactose.

Lactose derivatives of chitosan, indicated as CTL as a short name (previously reported in the literature as Chitlac), have recently emerged as intriguing examples of engineered biopolymers for potential applications in the field of drug delivery and tissue engineering [153]. From the chemical point of view, CTL is obtained through a *N*-alkylation reaction, which involves the amino groups of chitosan (Scheme 1).

The introduction of lactitol side chains on the chitosan backbone markedly modifies the physical-chemical properties of the polysaccharide rendering it soluble at neutral pH values and surprisingly miscible with polyanions, such as hyaluronan and alginate [154]. In the latter case, the interactions between the positive charges on CTL and the negative ones on alginate leads to the formation, under controlled conditions, of soluble complexes which determine a synergistic behavior both in dilute and semi-dilute conditions [155,156]. CTL was also used for the preparation of ternary mixtures with both alginate and hyaluronan [157,158]. A study of the conformational properties of CTL has been performed by means of NOE-NMR and molecular dynamics which showed a high flexibility of the lactitol side chain when compared to the chitosan backbone [159]. In addition, the lactitol side chain seems to adopt two distinct conformational orientations with respect to the polysaccharide backbone: a folded conformation and an extended one. In both conformations the side chains are well immersed in the aqueous solvent, showing a good degree of solvation. Molecular dynamics performed on decamers showed a propensity to form an extended 3_2_ helical geometry, but the preference shifted to the 2_1_ geometry with limited electrostatic interactions [160].

Leaving aside the physical-chemical properties, Chitlac has raised interest for its biological activity towards different primary cultures and cell lines. In particular, Chitlac promoted the aggregation of articular chondrocytes through the involvement of Galectin-1, [161,162] stimulating the production of collagen and glycosaminoglycans within an otherwise inert 3D architecture [163]. Similar biological activity has also been noticed on an osteoblast-like cell line [164]. Recently, cell aggregation promoted by lactose derivatives of chitosan was a prerequisite for the commitment of adipose-derived stem cells to the chondrogenic lineage [165]. Along this line, a lactose-modified chitosan was used to develop a hyaluronic acid-based hydrogel inducing stem cell aggregation during differentiation [165]. In addition, Chitlac was able to promote neuronal growth, differentiation, maturation, and formation of synapses, thus representing a good candidate for the development of complex bio-constructs for central nervous system regeneration [166].

Recently, a peculiar response of Chitlac-based macro-gels was noticed in the presence of a transient crosslinker, such as boric acid. In particular, shear-thickening and strain hardening effects of the chitlac-boric acid system grant it a mechanical response which is very similar to the one found for natural components of the extracellular matrix, such as collagen and neurofilaments [167,168]. This might disclose a new role for Chitlac in the field of biomaterials design and its involvement in mechanotransduction [169].

Moreover, Chitlac was found to form nano-gels in the presence of TPP as a crosslinker [153]. Resulting colloids were found to be highly monodisperse but, at the same time, poorly stable in physiological conditions of ionic strength, pH and temperature. Some of aforementioned limitations have been recently solved by reducing the amount of TPP, correspondingly replacing with low molecular weight hyaluronan [170]. The use of the anionic polysaccharide allowed for the synthesis of spherical-like coacervates, which proved to be stable against dissolution, behaving as pH-responsive carriers, thus, suitable for potential candidates in drug delivery.

## 11. Outlook

In this review we have summarized the main gelling concepts for synthesizing physical chitosan gels with different sizes and geometries. Key polymer features, such as the frequency of two building sugars—i.e., glucosamine and *N*-acetyl-glucosamine—and molecular weight are pivotal for the setting up of ordered networks. In addition, polymer concentration and external variables, such as temperature, pH, ionic strength, and crosslinker to chitosan monomer ratio, are in the same way fundamental for achieving a homogeneous gelation. It is possible nowadays to switch from a stable ensemble of sphere-like micro/nano-colloids to macrostructures by varying the aforementioned parameters. Resulting chitosan-based systems find a wide range of applications in the biomedical sector, spanning from the encapsulation and ensuing delivery of molecule therapeutics to their use as suitable scaffolds for cellular colonization.

In view of properly optimizing and customizing chitosan gels, chemical modifications of the parent biopolymer are often required. A lactose-modified chitosan named Chitlac is currently under investigation in our research group, since it showed intriguing biological features. Recent research records have highlighted the feasibility of forming Chitlac-based networks characterized by intriguing physical properties. One of the striking findings addressed the need to create a mechano-responsive material akin to what known for proteins composing the extracellular matrix (ECM). These results pave the way for a potential usage of Chitlac as a biomimetic ECM-substitute.
